# Supplementary material for: Drying kinetics and thermo-environmental analysis of a PV-operated tracking indirect solar dryer for tomato slices
Source: PLoS One. 2024 Oct 15;19(10):e0306281. doi: 10.1371/journal.pone.0306281 (PMC11478864; doi:10.1371/journal.pone.0306281)
Supplement: Attachment — Submitted filename: comments.pdf [file pone.0306281.s001.pdf]

List of comments

The article "Drying Kinetics and Thermo-Environmental of a PV-Operated Tracking Indirect Solar Dryer for Tomato Slices" has been reviewed, and the following recommendations are made for its improvement.

1) Select proper keywords.,i.e replace tomato fruit with tomato slice
2) Drying time (DT) did not significantly change with increasing air speeds from 1 to 2 m/s.Justify it in the abstract.
3) In recent years, several researchers have used mathematical models for studying the DK of many agricultural products such as magical berries [45], figs [46], parboiled rice [47], pistachio nuts [48], olives [49], eggplant [50], tomato [51], [52], green peppers [53], strawberries [54], sweet potato and raisins [55], seedless grapes [56], pears [46], green peas [57], pistachio kernels [58], peach [59], orange peel [60], cassava [61], peach slices [62], mango [63], and grapes [64], where they were investigated and researched drying under thin layer (ThL) conditions

How your model is different from these models, justify it, and introduce a paragraph after ref.[64]

4) Expand section 2: Materials and methods
5) Expand section 1.1.4 by adding about "Non-linear regression analysis"
6) Explain figs.1 to 8 in a separate section.
7) Role of your model in reducing energy crisis and co_2 emission.
8) Add some latest references in the introduction part for the years 2022-2023 including https://www.sciencedirect.com/science/article/abs/pii/S136403212300713X
9) Compare the Energy payback time (EPT) of your model with other similar models
10) Write the role of AI in solar dryers for future studies. This can be added in the conclusion for future scope
11) Overall presentation of the article is Good.
