# Supplementary material for: Drying kinetics and thermo-environmental analysis of a PV-operated tracking indirect solar dryer for tomato slices
Source: PLoS One. 2024 Oct 15;19(10):e0306281. doi: 10.1371/journal.pone.0306281 (PMC11478864; doi:10.1371/journal.pone.0306281)
Supplement: Attachment — Submitted filename: Response to reviewers for plos one journal (1).docx [file pone.0306281.s002.docx]

****Technical response to the reviewers****	May 5^th^, 2024

**Journal name: PLOS ONE**

**No.: PONE-D-24-12484**

**OLD Title:** “**~~Drying Kinetics and Thermo-Environmental of a PV-Operated Tracking Indirect Solar Dryer for Tomato Slices~~”**

**NEW Title: “Drying Kinetics and Thermo-Environmental Analysis of a PV-Operated Tracking Indirect Solar Dryer for Tomato Slices”**

**Abdallah E. Elwakeel^1^****^&*^, Mohsen A. Gameh^2^, Ahmed S. Eissa^3^, I. M. Elzein^4^, Mohamed Metwally Mahmoud^5^, Daniel Eutyche Mbadjoun Wapet^6&*^, Mahmoud M. Hussein^5,7^, Hany S. Hussein^8,9^, Aml Abubakr Tantawy^10^, Mostafa B. Mostafa^1^, Khaled A. Metwally^11^**

^1^ Agricultural Engineering Department, Faculty of Agriculture and Natural Resources, Aswan University, Aswan 81528, Egypt, [Abdallah_elshawadfy@agr.aswu.edu.eg](mailto:Abdallah_elshawadfy@agr.aswu.edu.eg); [Mostafabadawy017@gmail.com](mailto:Mostafabadawy017@gmail.com)

^2^ Soils and Water Department, Faculty of Agriculture, Assiut University, Assiut 71526, Egypt; [ali28@aun.edu.eg](mailto:ali28@aun.edu.eg)

^3^Agricultural Products Process Engineering Department, Faculty of Agricultural Engineering, Al-Azhar University, Cairo 11751, Egypt; [Ahmedeissa2205.el@azhar.edu.eg](mailto:Ahmedeissa2205.el@azhar.edu.eg)

^4^Department of Electrical engineering, University of Doha for Science and Technology, Doha, Qatar

60101973@udst.edu.qa

^5^Electrical Engineering Department, Faculty of Energy Engineering, Aswan University, Aswan 81528, Egypt, [Metwally_M@aswu.edu.eg](mailto:Metwally_M@aswu.edu.eg)

*^6&*^* National Advanced School of Engineering, Universit´e de Yaound´e I, Yaound´e, Cameroon, [eutychedan@gmail.com](mailto:eutychedan@gmail.com)

^7^Department of Communications Technology Engineering, Technical College, Imam Ja’afar Al-Sadiq University, Baghdad, 10053, Iraq, mahmoud_hussein@aswu.edu.eg

^8^Electrical Engineering Department, College of Engineering, King Khalid University, Abha 62529, Saudi Arabia

^9^Electrical Power Engineering Department, Faculty of Engineering, Aswan University, Aswan 81528, Egypt,

[hany.hussein@aswu.edu.eg](mailto:hany.hussein@aswu.edu.eg)

^10^Food Science and Technology Department, Faculty of Agriculture and Natural Resources, Aswan University, Aswan 81528, Egypt, [Aml.abubakr@agr.aswu.edu.eg](mailto:Aml.abubakr@agr.aswu.edu.eg)

^11^Soil and Water Sciences Department, Faculty of Technology and Development, Zagazig University,
Zagazig 44511, Egypt; [khametwally@zu.edu.eg](mailto:khametwally@zu.edu.eg)

*Corresponding author: Daniel Eutyche Mbadjoun Wapet, [eutychedan@gmail.com](mailto:eutychedan@gmail.com)

**Dear Editors and Reviewers**

The authors are thankful to the learned Editors and Reviewers for their thoughtful and detailed comments to improve the quality of the manuscript. The authors have tried to address all the concerns, and the corrections are incorporated in the revised manuscript. The replies to the reviewer’s comments are provided below.

We hope that this revised version can meet the reviewer’s expectations and the standards for publication in **PLOS ONE Journal.**

The changes incorporated in the revised manuscript are highlighted in YELLOW.

**Editor's Comments:**

Our sincere thanks and appreciation to the Editors for recommending the submission of the revised manuscript with major revision. To improve the quality of the manuscript, the reviewer's queries are addressed, and their suggestions are incorporated into the revised manuscript.

**Reviewer Comments:**

**Reviewer 1:**

**Comments to the Authors:**

**Comments1:  *Select proper keywords.,i.e replace tomato fruit with tomato slice***

**Response1:** The authors are extremely thankful to the reviewer for this thoughtful point. We agree with you, and we corrected it, kindly check the updated paper (***keywords***).

**Comment 2:  *Drying time (DT) did not significantly change with increasing air speeds from 1 to 2 m/s. Justify it in the abstract.***

**Response 2:** The authors are extremely thankful to the reviewer for this thoughtful point. We agree with you, and we added it, kindly check the updated paper (***Abstract***).

**Comment 3**:  *In recent years, several researchers have used mathematical models for studying the DK of many agricultural products such as magical berries [45], figs [46], parboiled rice [47], pistachio nuts [48], olives [49], eggplant [50], tomato [51], [52], green peppers [53], strawberries [54], sweet potato and raisins [55], seedless grapes [56], pears [46], green peas [57], pistachio kernels [58], peach [59], orange peel [60], cassava [61], peach slices [62], mango [63], and grapes [64], where they were investigated and researched drying under thin layer (ThL) conditions. How your model is different from these models, justify it, and introduce a paragraph after ref. [64]*

**Response 3:** The authors are extremely thankful to the reviewer for this thoughtful point. We agree with you, and we added it, kindly check the updated paper (***after Ref. 74***).

**Comment 4:**  Expand section 2: Materials and methods

**Response 4:** The authors are extremely thankful to the reviewer for this thoughtful point. The authors completely agree with you, but as explained in subtitle 2.2. in the updated paper, this study is complementing a previous study by Elwakeel et al. [16]

**Ref.** *Elwakeel, A. E., Mostafa B. Mostafa, Mohsen A. G., and Ahmed S. E. "Some Engineering Factors Affecting Utilization of Solar Energy in Drying Tomato Fruits." Aswan University Journal of Environmental Studies 5, no. 1 (2024): 52-68.*

**Comment 5:**  Expand section 1.1.4 by adding about “Non-linear regression analysis”.

**Response 5:** The authors are extremely thankful to the reviewer for this thoughtful point. The authors completely agree with you, and we added it, kindly check the updated paper (***page 7***).

**Comment 6:**  Explain figs.1 to 8 in a separate section.

**Response 6:** The authors are extremely thankful to the reviewer for this thoughtful point. We completely agree with you, but we have written the paper depends on the PLOS ONE journal format.

**Comment 7:**  Role of your model in reducing energy crisis and CO_2_ emission.

**Response 7:** The authors are extremely thankful to the reviewer for this thoughtful point. The role of the solar dryer for reducing or mitigation energy and CO_2_ per lifetime. **The annual thermal output** of both solar dryer and PV system was calculated and showed that values was 375.34, 399.23, and 446.86 kW.h for the solar dryer integrated with FCM, and was 423.74, 422.88, and 474.85 kW.h for the solar dryer integrated with TCM at ST of 8, 6, and 4 mm, respectively. **As well, CO_2_ per lifetime** was 5334.9, 5822.8 and 6795.4 tons for FCM and 6323.3, 6305.7 and 7366.9 tons for TCM, at tomato slice thicknesses of 8, 6, and 4 mm, respectively. Kindly check the updated paper (***Table 5 &*** page 20).

**Comment 8:**  Add some latest references in the introduction part for the years 2022-2023 including <https://www.sciencedirect.com/science/article/abs/pii/S136403212300713X>

**Response 8:** The authors are extremely thankful to the reviewer for this thoughtful point. We completely agree with you, and we added many recent paper most of them in the years 2023 & 2024, kindly check the updated paper.

**Comment 9:**  Compare the Energy payback time (EPT) of your model with other similar models.

**Response 9:** The authors are extremely thankful to the reviewer for this thoughtful point. We completely agree with you, kindly check the updated paper (Table 6).

**Comment 10:**  rite the role of AI in solar dryers for future studies. This can be added in the conclusion for future scope.

**Response 10:** The authors are extremely thankful to the reviewer for this thoughtful point. We completely agree with you, and we added the role of AI in solar dryers for future studies. Kindly check the updated paper (Conclusion and future work).

**Comment 11:**  Overall presentation of the article is Good.

**Response 11:** The authors are thankful to the honorable reviewer for the words of encouragement and trust in our work.

**Reviewer 2:**

**The authors are thankful to the honorable reviewer for the words of encouragement and trust in our work.**

**Comment-1: *What's the innovation of the paper? tomato slices drying kinetices? A lot of published papers have address the kinetics, so what's new? The highlights or innovations should be given in the section of introduction.***

**Response-1:** The authors are extremely thankful to the reviewer for this thoughtful point. We completely agree with you, and we added it to the introduction section, kindly check the updated paper.

**Comment-2: *If the the authors wants to do some contrast on the two dryer (tracking and no tracking), the performance should be investigated. But the author just give some drying kinetics, which is not related to dryer too much.***

**Response-2:** The authors are extremely thankful to the reviewer for this thoughtful point. The authors completely agree with you, but as explained in subtitle 2.2. in the updated paper, this study is complementing a previous study by Elwakeel et al. [16]

**Ref.** *Elwakeel, A. E., Mostafa B. Mostafa, Mohsen A. G., and Ahmed S. E. "Some Engineering Factors Affecting Utilization of Solar Energy in Drying Tomato Fruits." Aswan University Journal of Environmental Studies 5, no. 1 (2024): 52-68.*

While the current study was undertaken to discern the drying performance by utilizing both solar dryers integrated with FCM and TCM systems. By fitting drying curves with well-known models, the best model can be determined. Furthermore, calculate mathematical modeling. Finally, an environmental and energy analysis was performed to determine the energy payback time, and net CO_2_ mitigation over the lifetime of the developed solar dryer. Through this study, we hope to add to the body of knowledge already available on the drying kinetics of tomato fruit varieties and shed light on whether using solar dryers for this purpose would be environmentally feasible.

**Comment-3: *In the section of conclusion, please give more scientific evaluation.***

**Response-3:** The authors are extremely thankful to the reviewer for this thoughtful point. We agree with you, so we developed the conclusion section. Kindly check the updated paper (conclusion section).

**The authors once again thank the learned Editors and Reviewers for their valuable comments for improving the quality of the manuscript.**
