# Supplementary material for: Drying kinetics and thermo-environmental analysis of a PV-operated tracking indirect solar dryer for tomato slices
Source: PLoS One. 2024 Oct 15;19(10):e0306281. doi: 10.1371/journal.pone.0306281 (PMC11478864; doi:10.1371/journal.pone.0306281)
Supplement: Attachment — Submitted filename: Response to reviewers for plos one journal RR3.docx [file pone.0306281.s003.docx]

****Technical response to the reviewers****	Jun 11^th^, 2024

**Journal name: PLOS ONE**

**No.: PONE-D-24-12484**

**Drying Kinetics and** Thermo**-Environmental Analysis** of a PV-Operated Tracking Indirect Solar Dryer for Tomato Slices

**Abdallah Elshawadfy Elwakeel^1^****^&*^, Mohsen A. Gameh^2^, Awad Ali Tayoush Oraiath^3^, I. M. Elzein^4^, Ahmed S. Eissa^5^, Mohamed Metwally Mahmoud^6^, Daniel Eutyche Mbadjoun Wapet^7&*^, Mahmoud M. Hussein^6,8^, Aml Abubakr Tantawy^9^, Mostafa B. Mostafa^1^, Khaled A. Metwally^10^**

^1^ Agricultural Engineering Department, Faculty of Agriculture and Natural Resources, Aswan University, Aswan 81528, Egypt, [Abdallah_elshawadfy@agr.aswu.edu.eg](mailto:Abdallah_elshawadfy@agr.aswu.edu.eg); [Mostafabadawy017@gmail.com](mailto:Mostafabadawy017@gmail.com)

^2^ Soils and Water Department, Faculty of Agriculture, Assiut University, Assiut 71526, Egypt; [ali28@aun.edu.eg](mailto:ali28@aun.edu.eg)

^3^ Department of Agricultural Engineering, Faculty of Agriculture, Omar Al Mukhtar University, Libya 991, [awad.ali@omu.edu.ly](mailto:awad.ali@omu.edu.ly)

^4^Department of Electrical engineering, University of Doha for Science and Technology, Doha, Qatar

[60101973@udst.edu.qa](mailto:60101973@udst.edu.qa)

^5^Agricultural Products Process Engineering Department, Faculty of Agricultural Engineering, Al-Azhar University, Cairo 11751, Egypt; [Ahmedeissa2205.el@azhar.edu.eg](mailto:Ahmedeissa2205.el@azhar.edu.eg)

^6^Electrical Engineering Department, Faculty of Energy Engineering, Aswan University, Aswan 81528, Egypt, [Metwally_M@aswu.edu.eg](mailto:Metwally_M@aswu.edu.eg)

*^7&*^* National Advanced School of Engineering, Universit´e de Yaound´e I, Yaound´e, Cameroon, [eutychedan@gmail.com](mailto:eutychedan@gmail.com)

^8^ Department of Communications Technology Engineering, Technical College, Imam Ja’afar Al-Sadiq University, Baghdad, 10053, Iraq, mahmoud_hussein@aswu.edu.eg

^9^ Food Science and Technology Department, Faculty of Agriculture and Natural Resources, Aswan University, Aswan 81528, Egypt, [Aml.abubakr@agr.aswu.edu.eg](mailto:Aml.abubakr@agr.aswu.edu.eg)

^10^ Soil and Water Sciences Department, Faculty of Technology and Development, Zagazig University,
Zagazig 44519, Egypt; [khametwally@zu.edu.eg](mailto:khametwally@zu.edu.eg)

***Corresponding author:** Daniel Eutyche Mbadjoun Wapet, [eutychedan@gmail.com](mailto:eutychedan@gmail.com)

**Dear Editors and Reviewers**

The authors are thankful to the learned Editors and Reviewers for their thoughtful and detailed comments to improve the quality of the manuscript. The authors have tried to address all the concerns, and the corrections are incorporated in the revised manuscript. The replies to the reviewer’s comments are provided below.

We hope that this revised version can meet the reviewer’s expectations and the standards for publication in **PLOS ONE Journal.**

The changes incorporated in the revised manuscript are highlighted in YELLOW.

**Editor's Comments:**

Our sincere thanks and appreciation to the Editors for recommending the submission of the revised manuscript with major revision. To improve the quality of the manuscript, the reviewer's queries are addressed, and their suggestions are incorporated into the revised manuscript.

**Reviewer Comments:**

**Reviewer 2:**

**Comment-1: In the section of 2.6 data measuring, it's not so important on the error. According to the error propagation theory, what's the error of MR, and EE?**

**Response-1:** **The authors are extremely thankful to the reviewer for this thoughtful point. The error of MR, and EE were added to the illustrated data in Table 2. Kindly check the developed manuscript.**

**Comment-2: The results in Table 4 are the same with the Fig, 6 and 7? if so, I think you can choose one kind of them.**

**Response-2: The authors are extremely thankful to the reviewer for this thoughtful point. Where we deleted these figures. Kindly check the developed manuscript.**

**Comment-3: Especailly, what's the advantage using the kind of dryer? save energy? what should be highlighten.**

**Response-3:** **The authors are extremely thankful to the reviewer for this thoughtful point.**

**The solar dryer with a solar tracking collector offers significant advantages in terms of energy collected compared to the other solar dryer with a fixed collector:**

- **Increased Solar Exposure: The primary benefit is that a tracking collector can continuously orient itself towards the sun throughout the day. This maximizes the amount of solar radiation hitting the collector, leading to a significant increase in the heat energy collected. Fixed collectors, on the other hand, can only capture optimal sunlight for a limited period, typically around midday.**
- **Improved Drying Efficiency: With more collected heat energy, a tracking collector solar dryer can achieve faster drying times and higher drying capacity. This translates to improved overall drying efficiency.**
- **Reduced Drying Time: Faster drying times minimize the risk of spoilage for products being dried, especially food items. This is crucial for preserving the quality of the dried goods.**
- **Potential for year-round operation: Depending on the location and climate, a tracking collector can potentially extend the operational season of the solar dryer. By capturing more sunlight even during off-peak sun hours, tracking collectors can be more effective in areas with shorter daylight periods.**

**The authors once again thank the learned Editors and Reviewers for their valuable comments for improving the quality of the manuscript.**
